# Proteomic Analysis of the Fish Pathogen *Vibrio ordalii* Strain Vo-LM-18 and Its Outer Membrane Vesicles

**DOI:** 10.3390/ani14243598

**Published:** 2024-12-13

**Authors:** Macarena Echeverría-Bugueño, Mauricio Hernández, Ruben Avendaño-Herrera

**Affiliations:** 1 Interdisciplinary Center for Aquaculture Research (INCAR), Universidad Andrés Bello, Viña del Mar 2531015, Chile; mv.echeverriab@gmail.com; 2 Laboratorio de Patología de Organismos Acuáticos y Biotecnología Acuícola, Facultad de Ciencias de la Vida, Universidad Andrés Bello, Viña del Mar 2531015, Chile; 3 Division of Biotechnology, MELISA Institute, San Pedro de la Paz, Concepción 4133515, Chile; mhernandez@melisainstitute.org; 4 Centro de Investigación Marina Quintay (CIMARQ), Universidad Andrés Bello, Quintay 2480055, Chile

**Keywords:** vibriosis, proteome, salmonids, OMVs

## Abstract

*Vibrio ordalii* is the causative agent of atypical vibriosis in salmonids cultured in Chile. While extensive research provides insights into *V. ordalii* through phenotypic, antigenic, and genetic typing, as well as various virulence mechanisms, proteomic characterization remains largely unexplored. This study aimed to advance the proteomic knowledge of Chilean *V. ordalii* Vo-LM-18 and its OMVs, which have known virulence. Using Nano-UHPLC-LC-MS/MS, we identified 2242 proteins and 1755 proteins in its OMVs. Of these, 644 unique proteins were detected in *V. ordalii* Vo-LM-18, namely 156 unique proteins in its OMVs and 1596 shared proteins. The major categories for the OMVs were like those in the bacteria (i.e., cytoplasmic and cytoplasmic membrane proteins). Functional annotation identified 37 biological pathways in *V. ordalii* Vo-LM-18 and 28 in its OMVs. Proteins associated with transport, transcription, and virulence were predominant in both. Evident differences in protein expression were found. OMVs expressed a higher number of virulence-associated proteins, including those related to iron- and heme-uptake mechanisms. Notable pathways in the bacteria included flagellum assembly, heme group-associated proteins, and protein biosynthesis. This proteomic analysis is the first to detect the RTX toxin in a *V. ordalii* strain (Vo-LM-18) and its vesicles. Our results highlight the crucial role of OMVs in the pathogenesis and adaptation of *V. ordalii*, suggesting use as potential diagnostic biomarkers and therapeutic targets for bacterial infections.

## Simple Summary

This study investigates the set of proteins that comprise a *Vibrio ordalii* strain Vo-LM-18 and its outer membrane vesicles (OMVs), which are involved in the pathogen’s interaction with fish hosts, especially salmonids. *Vibrio ordalii* is a major cause of vibriosis, a disease that results in significant mortality in fish farms, a relevant industry in Chile. By analyzing the proteins expressed by both the bacteria and their OMVs, this research identifies key proteins linked to virulence, iron uptake, and cellular communication. The findings suggest that OMVs carry virulence factors that could aid in the pathogen’s ability to infect and persist in fish. These vesicles may also contribute to the pathogen’s survival in harsh environments, such as during host immune responses. This study provides new insights into bacterial pathogenesis and highlights potential targets for developing treatments or vaccines to combat infections in aquaculture.

## 1. Introduction

*Vibrio ordalii*, formerly *Vibrio anguillarum* biovar II [1], is the causative agent of atypical vibriosis, a hemorrhagic septicemia in several fish species, mainly in salmonids [2]. This bacterium is a Gram-negative, motile, rod-shaped microorganism that ferments glucose and oxidase, is catalase positive, and is sensitive to the vibriostatic agent O/129 [3]. Since 2004, *V. ordalii* has been reported as responsible for outbreaks with high mortalities in populations of Atlantic salmon (*Salmo salar*), Pacific salmon (*Oncorhynchus kisutch*), and rainbow trout (*Oncorhynchus mykiss)* cultured in southern Chile [4,5]. Fish affected by *V. ordalii* show necrosis and hemorrhagic lesions in the tissues surrounding the infection sites, including the ventral fin and anal pore [6].

Despite *V. ordalii* and *V. anguillarum* being very closely related genomically, especially the serotype O2 isolates [7,8], until now, most research has been carried out to elucidate the virulence factors and pathogenesis of *V. anguillarum* [7,9]. In the case of *V. ordalii*, the main studies have focused on phenotypic, serotype, and genetic differences among isolates [1,4,5,10], but pathogenesis is not fully understood. This notwithstanding, properties for adhesion, colonization, and invasion [11]; exotoxins [6]; cell-surface components or capsular material [11,12]; and iron-uptake systems [13,14,15] have been reported. All of these features have been confirmed by the presence of numerous likely genes in the genome of the strain type *V. ordalii* ATCC 33209^T^ [7].

More recently, we delved into some characteristics that could be involved in the virulence mechanisms of *V. ordalii*; in vitro findings support the facultative intracellular behavior of this pathogen [16] and have demonstrated that *V. ordalii* produce and release outer membrane vesicles (OMVs) under normal growth conditions [17]. In addition, purified OMVs demonstrate hemolytic enzyme activity, while protein analysis has detected a 38 kDa protein that would correspond to the OmpU protein found in the membrane for *V. anguillarum* [18], which is associated with pathogen adaptation to the host, increasing resistance to bile and favoring biofilm formation during *V. anguillarum* infection [19].

Little to no information is available regarding the proteomic characterization of *V. ordalii*. Therefore, the aim of the present study was to further proteomic knowledge of the Chilean *V. ordalii* Vo-LM-18 and its vesicles. This strain has been broadly characterized by our team, and our research constitutes the first report of a proteomic approach for studying this fish pathogen.

## 2. Materials and Methods

### 2.1. V. ordalii Growth

In this study, the *V. ordalii* strain Vo-LM-18 and its OMVs were employed. This strain was originally isolated from a vibriosis outbreak in Atlantic salmon, and different characteristics associated with its virulence have been studied by our research group (e.g., cell-surface properties and iron-uptake mechanisms, among others) [11,15]. In addition, strain Vo-LM-18 was selected due to the previous characterization of OMVs production [17]. Standard phenotypical procedures [5] and a previously described PCR protocol [20] confirmed strain Vo-LM-18 as *V. ordalii*. The bacterium was routinely cultivated under aerobic conditions at 18 °C for 48 h in trypticase soya agar or broth (BD) with 1% (*w*/*v*) NaCl (Winkler) (TSA-1 and TSB-1, respectively). Stock cultures were kept frozen at −80 °C in Cryobank tubes (Mast Group, Liverpool, UK) or in TSB-1 with 15% (*v*/*v*) glycerol.

### 2.2. V. ordalii Cells and OMVs Isolation

The *V. ordalii* cells and OMVs were obtained from *V. ordalii* grown in a liquid culture. Once the Vo-LM-18 colonies were grown, three pure colonies were used to prepare the starting inocula in tubes containing 5 mL of TSB-1. Bottles with 800 mL of culture medium were finally seeded with each inoculum of *V. ordalii* until a concentration of 0.2 at 620 nm in a NanoQuant Microplate Spectrophotometer (Tecan, Seestrasse, Switzerland) was reached. All experiments were performed in triplicate. Then, *V. ordalii* cells were removed by centrifugation (10,000× *g* for 15 min at 4 °C) and washed four times with phosphate-buffered saline 1X (pH 7.4) at 5000× *g* for 10 min at 4 °C to remove broth remnants. Then, each cellular pellet was frozen at −80 °C until use.

The purity of strain Vo-LM-18 after centrifugation was confirmed by Gram staining, cell morphology, and PCR, as described above. The OMVs were obtained exactly as described by Echeverría-Bugueño et al. [17]. The supernatant was filtered through a 0.45 µm and a 0.22 µm pore size consecutively (JETbiofill), and another round of ultracentrifugation was performed at 125,000× *g* for 2 h at 4 °C. The pellet was recovered and resuspended in milli-Q water with 1% protease inhibitor (Protease Inhibitor Cocktail Set I, Animal-Free—Calbiochem). To collect high-purity OMVs, a new round of ultracentrifugation for 60 min was performed, and the pellet was resuspended in milli-Q water with 1% protease inhibitor and stored at −80 °C. Furthermore, 5 µL of purified OMVs were grown in TSB-1 agar to confirm all cells were eliminated during the process.

### 2.3. Scanning Electron Microscopy (SEM)

SEM was prepared according to Echeverría-Bugueño et al. [17], with slight modifications. Bacteria and OMVs were fixed with 2.5% glutaraldehyde (Merck) in a 0.1 M sodium cacodylate buffer (Sigma, CB, pH 7.4) for 60 min at room temperature. The samples were deposited in a cover sheet treated with poly-L-lysine and dehydrated in a battery of alcohols. Then, critical-point drying was performed (Critical point dryer HCP-2, Hitachi, Tokyo, Japan). Unlike what was previously reported [17], we used coverslips instead of cooper grids. The coverslips were deposited on sample holders previously covered with carbon tape (EMS), and the samples were shaded with 1 nm gold palladium (Leica EM ACE 200, Wetzlar, Germany). The images were obtained using AURIGA Compact-Field scanning transmission electron microscopes (FESEM, STEM), a focus ion beam (FIB-SEM) at 5.0 kV, and a working distance between 4 and 5 nm. Finally, the sizes of the bacteria and OMVs were determined using the ImageJ software (NIH, https://imagej.nih.gov accessed on 20 June 2024).

### 2.4. Protein Extraction for nLC-MS/MS

Each replicate of the Vo-LM-18 cells and OMVs suspension was lyophilized overnight. Subsequently, each sample was resuspended in 500 µL of 8 M urea with 25 mM sodium bicarbonate and sonicated for 1 min with 3 pulses of 9 s at 40% intensity. Finally, the samples were centrifuged at 10,000× *g* for 10 min, discarding the pellet and storing the supernatant at −80 °C.

### 2.5. Protein Extraction and Digestion for nLC-MS/MS

The proteins of each sample were subjected to precipitation using 5:1 *v*/*v* cold acetone 100% and incubated overnight at −20 °C; then, they were centrifuged at 15,000× *g* for 10 min; the supernatant was discarded, and the pellet was washed three times with acetone at 90% *v*/*v*, dried in a rotary concentrator at 4 °C, and finally resuspended in 8 M urea with 25 mM of ammonium bicarbonate (NH_4_HCO_3_, pH 8.0). The proteins were quantified with a Qubit protein assay, where 100 µg were reduced with 20 mM dithiothreitol for 60 min, alkylated with 20 mM iodoacetamide in the dark for 60 min, diluted ten times with 25 mM of ammonium bicarbonate pH 8.0, and digested with trypsin/LyC (Promega, Madison, WI, USA) in a 1:50 ratio overnight at 37 °C. Peptides were cleaned using Pierce C-18 Spin Columns (Thermo Scientific, Waltham, MA, USA) according to the manufacturer’s instructions, and the eluted peptides were dried using a rotary concentrator at 4 °C, resuspended in 2% acetonitrile with 0.1% (*v*/*v*) formic acid (Merck, Darmstadt, Germany), and quantified using a Direct Detect Spectrometer (Merck Millipore, Darmstadt, Germany).

### 2.6. Liquid Chromatography

A nanoElute^®^ liquid chromatography system (Bruker Daltonics, Billerica, MA, USA) was used, and peptides (200 ng of digest) were separated within 90 min at a flow rate of 400 nL/min on a reversed-phase column Aurora Series CSI (25 cm × 75 µm i.d. C18 1.6 µm) (IonOpticks, Fitzroy, Australia) at 50 °C. Mobile phases A and B were water and acetonitrile with 0.1% (*v*/*v*) formic acid, respectively. The B percentage was linearly increased from 2 to 17% within 57 min, followed by an increase to 25% B within 21 min and further to 35% within 13 min, followed by a washing step at 85% B and re-equilibration.

### 2.7. The timsTOF Pro Mass Spectrometer

All samples were analyzed using three biological replicates per condition using the hybrid trapped ion mobility spectrometry (TIMS) quadrupole time-of-flight mass spectrometer (MS) (TIMS-TOF Pro, Bruker Daltonics) through a CaptiveSpray nano-electrospray ion source. The MS was operated in data-dependent mode for the ion mobility-enhanced spectral library generation. We set the accumulation and ramp time at 100 ms each and recorded mass spectra in the range from *m*/*z* 100–1700 in positive electrospray mode. Ion mobility was scanned from 0.6 to 1.6 Vs/cm^2^. The overall acquisition cycle of 1.16 s comprised one full TIMS-MS scan and ten parallel accumulation-serial fragmentation (PASEF) MS/MS scans.

### 2.8. Database Searching

Tandem mass spectra were extracted by TIMS Control v.2.0. Charge state deconvolution and deisotoping were not performed. All MS/MS samples were analyzed using PEAKS Studio (Bioinformatics Solutions, Waterloo, ON Canada; v10.5 (20 November 2019)) [21,22]. PEAKS Studio was set up to search the UniProt_SwissProt *Vibrio ordalii* database (3390 entries) assuming the digestion enzyme trypsin. PEAKS Studio was searched with a fragment ion mass tolerance of 0.050 Da and a parent ion tolerance of 50 PPM. Carbamidomethyl of cysteine was specified in PEAKS Studio as a fixed modification. The deamidation of asparagine and glutamine; the oxidation of methionine; acetyl of the n-terminus; and carbamoyl of lysine and the n-terminus were specified in PEAKS Studio as variable modifications.

### 2.9. Label-Free Quantitation and Differential Expression Analysis

Individual identification reports from PEAKS were concatenated and resulting missing values (NA) were imputed by MICE [23]. To determine which proteins were differentially and significantly expressed in the treatments’ contrast, we applied a Wald test to data with a Benjamini–Hochberg correction using Deseq2 [24]. Any protein associated with a *p*-adjust < 0.05 was considered significant. The identification of Differentially Expressed Proteins (DEPs) was carried out by comparing the expression ratio of proteins identified in OMVs versus bacteria.

Graphical representations related to quantification results were created using the statistical environment R [25] with EnhancedVolcano [26], Complex Heatmap v.2.0.0 [27], GOplot [28], and R base packages. In addition, the prediction of the subcellular localization of the proteins identified in OMVs and bacteria was carried out through PSORTb v3.0 using default parameters [29].

Sequence protein annotation was performed by Sma3s [30] with default parameters. The protein–protein interaction was carried out by mapping protein sequences against *Vibrio anguillarum* in the STRING database [31]. The resulting network was imported and processed in Cytoscape v.3.91 [32].

## 3. Results and Discussion

### 3.1. Purification of OMVs and Characterization of the Bacterial/OMV Proteome

**Figure 1:**
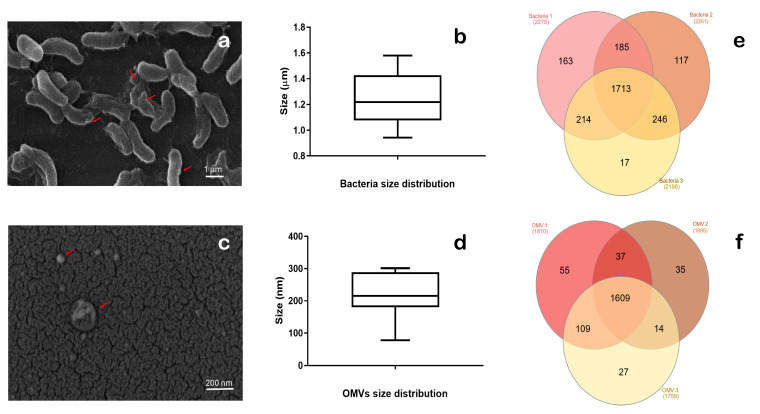
Characteristics of *Vibrio ordalii* Vo-LM-18 and its OMVs. SEM visualization of (**a**) the bacteria and (**c**) its OMVs. SEM-determined size of (**b**) the bacterium and (**d**) OMVs, showing greater heterogeneity in OMVs sizes vs. bacteria. Venn diagram illustrating proteins identified across each replicate of (**e**) *Vibrio ordalii* Vo-LM-18 and (**f**) its OMVs. The arrowhead points to the OMVs.

To confirm the purity of *V. ordalii* Vo-LM-18 cells and its OMVs after isolating each component, they were visualized using SEM. *V. ordalii* Vo-LM-18 cells exhibited the classic morphology of this species, i.e., bacillus shape with flagella (Figure 1a) and, with an average size of 1.24 ± 0.19 µm (Figure 1b), consistent with the values obtained in previous studies for this strain [17]. The bacteria, during exponential growth, constitutively released vesicles into the external medium (Figure 1a). Additionally, the SEM of the OMVs purified by ultracentrifugation revealed pure vesicles without the presence of bacteria or debris (Figure 1c), showing a heterogeneous distribution with closed elliptical and/or spherical shapes. The vesicles observed had an average size of 218.228 ± 62 nm with a range between 78 and 218.3 nm (Figure 1d). It is important to note that these OMVs were found in the crude extract of the *V. ordalii* culture, so our study did not select subpopulations based on sizes, as indicated by Crescitelli et al. [33].

To identify the proteome of *V. ordalii* Vo-LM-18 and its OMVs, peptides were quantified and analyzed using nanoscale high-performance liquid chromatography coupled to tandem mass spectrometry (nLC-MS/MS). Each experiment included three biological replicates per sample type (*V. ordalii* Vo-LM-18 and OMVs), showing very similar protein values in each case (Figure 1e,f). A total of 30,683.3 peptides were detected in the bacteria, resulting in 2242 ± 106 proteins (Figure 1e), whereas a total of 17,749 peptides were evaluated in the vesicles, resulting in 1754.7 ± 57 proteins (Figure 1f). This represented a reduction of 488 proteins in the vesicles compared to the bacteria from which they originated.

### 3.2. Cellular Distribution and Major Functions of the Proteome of V. ordalii and Its OMVs

**Figure 2:**
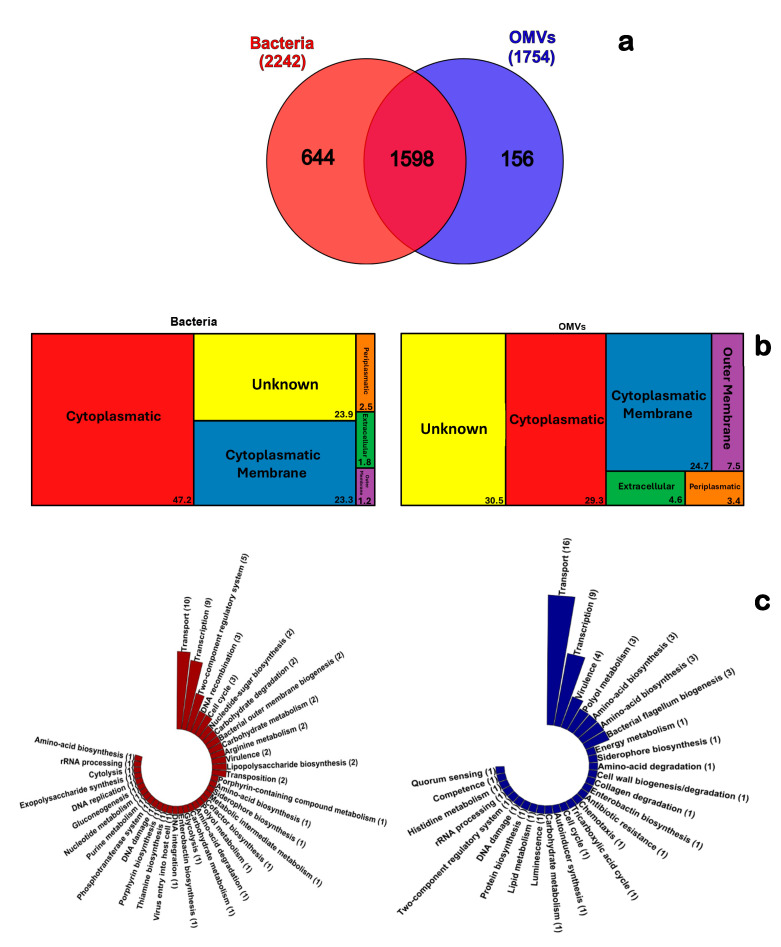
Proteomic and protein subcellular localization resources (PSORT) analyses of *Vibrio ordalii* strain Vo-LM-18 and its OMVs. (**a**) Venn diagram showing the overlap of proteins identified in the Vo-LM-18 strain and its OMVs. (**b**) Distribution of proteins by cellular compartment as predicted by PSORT. (**c**) Functional categorization and grouping of proteins annotated by Gene Ontology.

Of the proteins identified in *V. ordalii* Vo-LM-18 and its OMVs, 644 and 156 unique proteins were detected in each component, while 1596 proteins were shared (Figure 2a). Using the PSORT prediction tool, a compartmental distribution of the identified proteins was determined. In the case of strain Vo-LM-18, the most abundant category was cytoplasmic, comprising 47.2% of the proteins, followed by the cytoplasmic membrane with 23.3%. A similar percentage of proteins fell into the category of unknown localization (23.9%) (Figure 2b). The major categories in the OMVs were the same as those detected in the bacteria (Figure 2b), but with different percentages. Proteins in the unknown category were more abundant (30.5%), followed by the cytoplasmic category at 29.3%, and then the cytoplasmic membrane at 24.7%. Additionally, three other minor categories were detected —outer membrane, extracellular, and periplasmic—with contributions of 7.5%, 4.6%, and 3.4%, respectively. Interestingly, it was observed that in OMVs, the proteins of the outer membrane were more enriched compared to the bacteria, with 1.2% for *V. ordalii* and 7.5% for OMVs.

When performing analysis of functional annotations by Gene Ontology at the level of distribution into biological categories (Figure 2c), 37 and 28 biological pathways were detected in *V. ordalii* Vo-LM-18 and its OMVs, respectively. The proteins associated with transport, transcription, and virulence were predominant both in the bacteria and in the vesicles, while another 11 pathways were also shared, but with a value of 1 in the *V. ordalii* Vo-LM-18 strain or its OMVs. It is interesting to note that both contained proteins associated with siderophore biosynthesis, enterobactin biosynthesis, DNA damage, and rRNA processing. Specifically, piscibactin and vanchrobactin have been demonstrated as siderophores, along with several iron uptake mechanisms based on heme utilization in *V. ordalii* [14,15]. However, this was the first detection of proteins associated with enterobactin, a xenosiderophore originally described in *Escherichia coli* [34]. Its functional relationship with the siderophore vanchrobactin has been previously reported in *V. anguillarum* [35] and other pathogens of the *Vibrionaceae* family [36,37]. On the other hand, 15 out of the 28 biological pathways of the OMVs were not detected in the bacteria, including bacterial flagellum biogenesis, collagen degradation, luminescence, and quorum sensing. All these have been associated with virulence mechanisms within the *Vibrio* genus, especially in other fish pathogens [2,38,39].

### 3.3. Analysis of Differences and Similarities in the Proteome of V. ordalii Vo-LM-18 and Its OMVs

**Figure 3:**
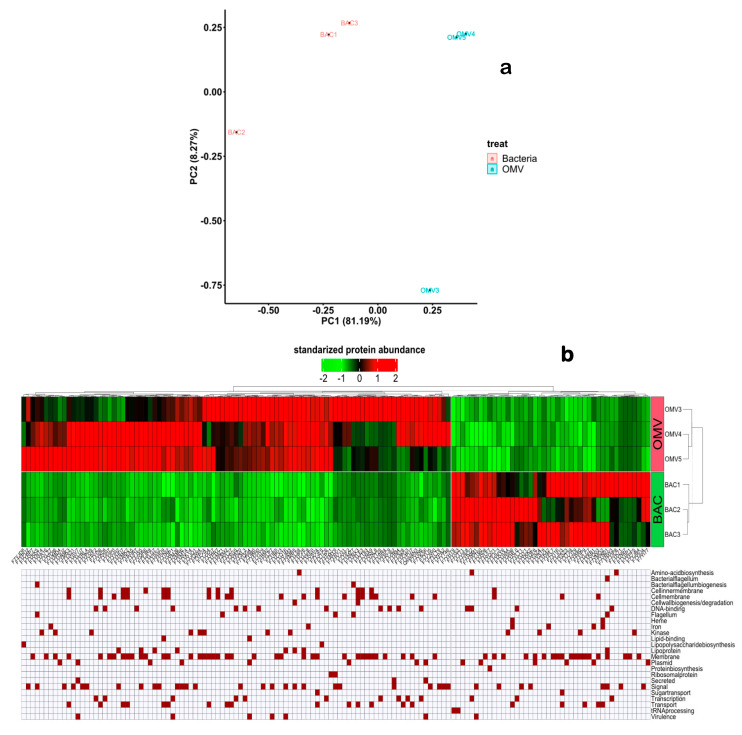
Graphic representation of the quantitative proteomic analysis of *Vibrio ordalii* Vo-LM-18 and its OMVs. (**a**) Principal component analysis (PCA) of quantifiable proteins from *Vibrio ordalii* Vo-LM-18 and its OMVs. (**b**) Heatmap displaying significantly differentially expressed proteins (DEPs) common to both the Vo-LM-18 strain and its OMVs, along with their functional distribution.

To assess differences between the proteomes of *V. ordalii* Vo-LM-18 and OMVs, we conducted a principal component analysis (PCA). PCA involves reducing the dimensionality of the data in an unsupervised manner, aiming to observe if each condition forms distinct groups and if they differ between conditions. Our analysis revealed well-defined clusters for *V. ordalii* Vo-LM-18 and its OMVs, with component 1 explaining 81.19% of the variance and component 2 explaining 8.27% (Figure 3a). This confirmed that the proteomes of *V. ordalii* Vo-LM-18 and its OMVs exhibited qualitative and quantitative differences, indicating distinct protein profiles.

The DEPs were represented on a heatmap (Figure 3b), which confirmed the homogeneity of the triplicates, and the robustness of the results obtained. The heatmap also displayed the functional annotation and pathways of some proteins, especially those considered relevant for bacteria belonging to the *Vibrio* genus, with an abundance criterion ranging from −2 to 2. When comparing between strain Vo-LM-18 and its OMVs, differences between both samples were evident, showing that the OMVs expressed a higher number of virulence-associated proteins, including iron- and heme- mechanisms related to membrane proteins (Figure 3b). Additionally, the OMVs contained proteins involved in flagellum biosynthesis, lipopolysaccharide synthesis, and membrane degradation, as well as signal-associated and lipid-binding proteins, ribosomal proteins, and secreted proteins. Regarding the predominant pathways in the bacteria, the highlighted proteins included those related to flagellum assembly, heme group-associated proteins, and those involved in protein biosynthesis.

### 3.4. Label-Free Quantitation of Common Proteins Between V. ordalii and Its OMVs

We carried out label-free quantification analysis using the OMVs versus bacteria relationship. A total of 211 statistically significant DEPs were obtained using a *p*-value of 0.05% as a cut-off point of statistical significance. Results obtained 72 under-expressed proteins and 139 over-expressed proteins, observing significant changes in the expression of proteins in the order of 10 Log2 fold change (FC) between OMVs versus *V. ordalii*. The protein that had a most dramatic difference in expression was an Iron (III) ABC transporter ATP-binding Protein hitC, with a more than a 9-fold change in the Log2FC under-expressed in OMVs with respect to *V. ordalii* Vo-LM-18. In turn, the transporter of toxin A was the second most over-expressed protein in OMVs (rtxB), at seven times the Log2FC. With respect to the bacteria, interestingly, toxin A (rtxA) was also over-expressed by at least four times the Log2FC. It is important to note that this RTX toxin has not been previously described in *V. ordalii*. However, it has been identified in other pathogenic species of the *Vibrio* genus, including *V. cholerae* [40], *V. vulnificus* [41], and *V. anguillarum* [42], with the latter being taxonomically close to *V. ordalii* [1]. Therefore, this proteomic analysis represents the first detection of the RTX toxin in a *V. ordalii* strain (Vo-LM-18) and its vesicles.

The two bacteria from the *Vibrio* genus in which the RTX toxins have been characterized in the greatest depth are *V. cholerae* and *V. vulnificus*. In both pathogens, classic functions associated with this group of toxins have been described, including participation in colonization, adherence to host epithelia, a role in virulence, and a protective role against the host phagocytosis response; interestingly, only *V. cholerae* had the capacity to generate actin dimers as a possible entry pathway to the host and of pathogenicity [41,43,44]. In the case described for *V. anguillarum*, a bacterium phylogenetically close to *V. ordalii*, one of the main virulence factors of the pathogen, such as hemolysis, is related to a multi-copy rtx operon that maintains activity even in the face of mutations [42]. In addition, in *V. anguillarum*, the relationship between stressors such as temperature and iron availability and RTX toxins has been described; at low temperatures (15 °C), the hemolysin RTX pore-forming toxin, T6SS2, increases its expression. This increase in T6SS2 expression goes hand in hand with the expression of iron uptake components, such as the siderophore piscibactin, so it also plays a role in the response to iron limitation [45]. Considering the present study is the first description in *V. ordalii* Vo-LM-18, in vitro assays are required to determine the influence of the toxins of the RTX family described in Table 1, not only in the virulence of the pathogen but also in its response to host immunity and environmental variations. In contrast, a flavohemoglobin protein related to the response to nitric oxide stress showed a 1.78-fold decrease in expression (Log2FC). This under-expression may give us an idea of the temporal role of OMVs. For example, upon infection of European seabass (*Dicentrarchus labrax*) with *V. anguillarum*, iNOS levels are increased at the mucosal level [46]. These prior findings suggest that OMVs are unable to trigger the anti-iNOS response at the mucosal level and would act at the level of early infection. Additionally, another group of four proteins associated with metabolism was observed. A selection of 54 proteins, with Log2(FC) values ranging from −1.78 to 9.27, included 37 over-expressed and 17 under-expressed proteins (Table 1).

Additionally, the bacteria exhibited an over-expression of proteins associated with metabolism, chemotaxis, and nutrient uptake. In terms of metabolism, the predominant protein identified was FlgH, which is associated with flagellar movement along with MutH, GspJ (equivalent to EspJ), and VipA/VipB [47,48,49,50]. For chemotaxis, the CheW protein and a transmembrane receptor were found [51]. Regarding nutrient and metal uptake, the identified proteins included the STAS domain associated with anion transporters, an ATP-dependent iron transporter, and phospholipases [52].

The over-expressed proteins in *V. ordalii* Vo-LM-18 OMVs included a variety of proteins that could contribute to their virulence and ability to survive in the host. For example, the presence of proteins such as TolQ (4.3-fold over-expressed), associated with iron uptake, suggests an important role in the acquisition of essential nutrients by the bacterium during infection [53,54]. In addition, proteins such as OmpA and OppA, known to be involved in host–cell attachment and antimicrobial resistance, respectively, indicate strategies that could help *V. ordalii* evade host defenses and persist in the hostile environment of the intestinal tract, as previously reported for *V. anguillarum*, *V. harveyi*, and *V. furnissii* [47,55,56].

Interestingly, many of the over-expressed proteins in *V. ordalii* Vo-LM-18 OMVs were associated with virulence and antimicrobial resistance, suggesting that these vesicles could play an important role in the spread of virulence factors and resistance genes within the bacterial population and between different hosts. Furthermore, the presence of proteins such as VgrG, associated with membrane perforation and phage infection [57,58,59], suggests that OMVs could be used as vehicles for the delivery of toxins and virulence factors directly into host cells, thereby enhancing the ability of the bacterium to colonize and cause disease.

The under-expressed proteins in *V. ordalii* Vo-LM-18 OMVs represented a selection of proteins related to different cellular functions, all of which could have implications for bacterial survival. For example, the presence of proteins associated with the replication of genetic material, such as MutH, indicates that the bacterium retains these proteins to maintain the integrity of its genome and ensure accurate replication during its lifecycle [50]. Similarly, proteins associated with chemotaxis, such as the methyl-accepting chemotaxis protein, suggest that the bacterium needs these proteins to move toward or away from certain environmental stimuli, which could be crucial to its ability to colonize and persist in the host [60].

Overall, these findings highlight the importance of OMVs in the pathogenesis of *V. ordalii* Vo-LM-18 and suggest that these vesicles could play a key role in the adaptation and survival of the bacterium in different environments, including the host and aquatic environment. However, further studies are needed to fully understand the role of OMVs in the biology and pathogenesis of *V. ordalii*, as well as their potential as therapeutic targets or biomarkers for the diagnosis of infection.

### 3.5. Analysis of Pathways and Co-Expression Through DEP Networks

**Figure 4:**
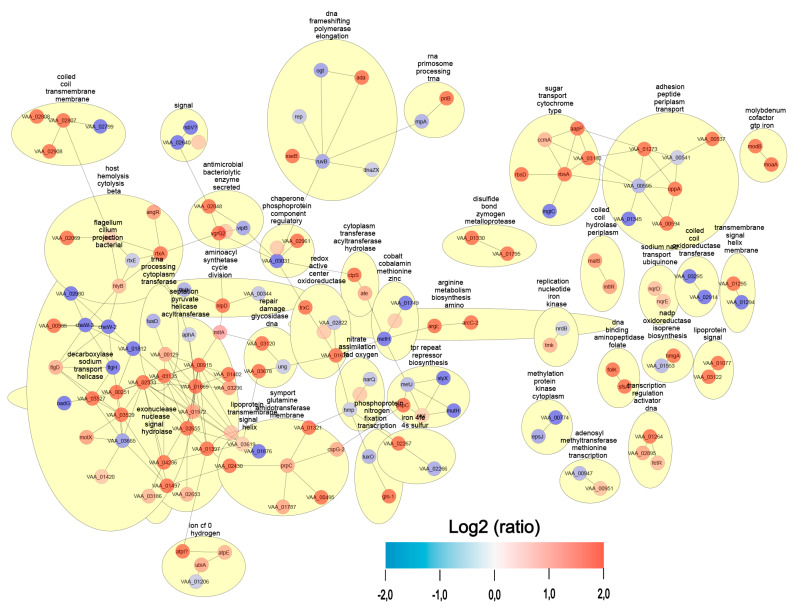
Co-expression networks in *Vibrio ordalii* Vo-LM-18 and its OMVs. Each node represents a metabolic pathway, and the edges between nodes indicate subordinate relationships between different pathways. Proteins involved in each pathway are highlighted as either over-expressed (red) or under-expressed (blue).

After the functional annotation of the proteins, we performed network analysis where changes were observed in different metabolic pathways related to primary metabolism and, in turn, to virulence factors (Figure 4). In fact, the co-expression networks in *V. ordalii* Vo-LM-18 versus OMVs further reinforced the observations made in the heatmap (Figure 4). For instance, pathways whose proteins were over-expressed to a greater extent were related to iron uptake and virulence factors such as exonucleases and hemolysins. In contrast, pathways whose proteins were under-expressed were associated with metabolic processes such as transcription, signaling, and the transport of molecules such as sodium (Figure 4). As seen, these results pointed to an increase in pathways related to bacterial virulence and invasion.

## 4. Conclusions

Our findings reveal significant differences between the proteomes of *V. ordalii* strain Vo-LM-18 and its OMVs, highlighting a higher abundance of virulence and transport proteins in OMVs. For example, the over-expression of proteins such as TolQ suggest a role in host–cell infection and virulence. In contrast, the under expression of proteins such as MutH in OMVs suggests adaptations in the survival strategy of *V. ordalii* by shifting roles associated, for example, with transcription. These results underscore the crucial role of OMVs in the pathogenesis and adaptation of *V. ordalii*, suggesting their potential as diagnostic biomarkers and therapeutic targets for bacterial infections.

## Figures and Tables

**Table 1:** Summary of the predicted over-expressed (DEPs+) and under-expressed (DEPs−) proteins. Fisher’s test with a *p*-value less than 0.05 was used as the cutoff for pathway enrichment.

Gene Name	Description	Molecular Function	LogFC
hitC	Iron(III) ABC transporter ATP-binding protein	Iron uptake	9.27
rtxB	RTX toxin transporter RtxB	Virulence	7.13
waaF	Heptosyltransferase II	Biosynthesis LPS	6.75
hap/vvp	Hemagglutinin/proteinase Hapa	Virulence	6.53
VAA_00552	Drug and metabolite transporter	Transport	6.50
vasL	Type VI secretion system protein VasL	Secretion system	5.71
letS	Hybrid sensor histidine kinasa/response regulator LetA/S	Regulation	5.44
VAA_02069	OmpF 100% homology with OmpA	Virulence	4.94
rtxA	RTX toxin RtxA	Exotoxin/Virulence	4.81
smcL	Exoenzyme sphingomyelinase-c	Virulence	4.72
vgrG-3	Type VI secretion system effector VgrG-3	Secretion system/Toxin	4.68
VAA_01805	Phospholipase	Colonization	4.54
VAA_01388	TolQ	Cell division	4.37
VAA_02430	Zinc protease	Pathogenicity/Virulence	4.32
msrA/B(pilB)	Peptide methionine sulfoxide reductase MsrA	Oxidative damage	4.25
VAA_01397	Peptidase domain M75 related to pili function	Proteolysis	4.23
VAA_02130	PspC	Shock response	4.21
flgM	Negative regulator of flagellin synthesis FlgM	Biosynthesis flagellin	3.97
lapA	Probable Lipopolysaccharide A assembly protein	Biosynthesis LPS	3.79
VAA_02321	Endochitinase	Nutrient uptake	3.67
acfB	Accessory factor AcfB for intestinal colonization	Colonization	3.27
per	Perosame synthetase from a unusual LPS sugar	Biosynthesis LPS	3.27
fbpC	Iron(III) ABC transporter ATP-binding protein	Iron uptake	3.26
VAA_03250	OppA	Uptake of peptides	3.16
flgP	Vibrio-specific flagellar H-ring component FlgP	Motility	3.04
entF	Enterobactin synthase, multienzyme complex component	Iron uptake	2.94
motX	Sodium-type flagellar protein MotX	Motility	2.74
VAA_02157	IpgD	Colonization	2.62
sopB/sigD	Type III secretion system effector SopB	Secretion system	2.62
VAA_02312	Glucosidase	Carbohydrates biosynthesis	2.58
flgD	Flagellar basal-body rod modification protein FlgD	Motility	2.33
irp1	Yersiniabactin biosynthetic protein Irp1	Iron uptake	2.23
ccmA	ABC transporter	Cytocromo c Biogenesis	2.18
bauE	Ferric siderophore ABC transporter protein BauE	Iron uptake	2.18
cdpA	Cyclic di-GMP phosphodiesterase CdpA	Regulation	2.16
tcpI	Negative regulator of the major pilin TcpA	Motility	2.02
ectA	L-2,4-Diaminobutyric acid acetyltransferase	Amino acid biosynthesis	1.70
flgH	Flagellar L-ring protein FlgH	Motility	−6.21
VAA-01413	STAS domain	Anion transporter	−6.15
tcpI	Regulator of the major pilin TcpA	Adherence	−5.66
mutH	MutH	DNA repair	−4.57
VAA_03003	ATP-dependent iron transport protein	Iron uptake	−4.20
VAA_03329	Phospholipase	Nutrient uptake/Virulence	−3.59
mam7	Multivalent adhesion molecule MAM7	Adherence	−3.50
VAA_01876	Paraquat-inducible B protein	Stress response	−3.50
cheW	Purine-binding chemotaxis protein CheW	Chemotaxis	−3.47
clpV1	Protein that recycles VipA/VipB tubules and prevents non-productive tubule formation	Secretion system	−3.46
VAA_02980	Chemotaxis methyl acceptor protein	Chemotaxis	−3.36
VAA_01345	NorM protein	Multidrug efflux	−3.30
epsJ	Type II secretion system minor pseudopilin GspJ	Motility	−2.98
fleQ	Pivotal role in promoting higher-order functional oligomers	Regulation	−2.57
pchA	Transmembrane signaling receptor activity	Chemotaxis	−1.89
cyaB	Cyclolysin secretion ATP-binding protein Cya	Exotoxin	−1.84
VAA_00966	Flavohemoglobin	Oxidative stress	−1.78

## Data Availability

The data are available upon request from the corresponding author.
